# Elicitation of gymnemic acid production in cell suspension cultures of *Gymnema sylvestre* R.Br. through endophytic fungi

**DOI:** 10.1007/s13205-016-0555-y

**Published:** 2016-10-28

**Authors:** Vasudeva Reddy Netala, Venkata Subbaiah Kotakadi, Susmila Aparna Gaddam, Sukhendu Bikash Ghosh, Vijaya Tartte

**Affiliations:** 1 Department of Biotechnology, Sri Venkateswara University, Tirupati, AP India; 2 DST-PURSE Center, Sri Venkateswara University, Tirupati, AP India; 3 Department of Virology, Sri Venkateswara University, Tirupati, AP India; 4 Nuclear Agriculture and Biotechnology Division, BARC, Mumbai, MH India; 5 Department of Botany, Sri Venkateswara University, Tirupati, AP India

**Keywords:** *Gymnema sylvestre*, Endophytic fungi, 18S rRNA, *Polyancora globos*a, Cell suspension cultures, Biotic elicitor, Gymnemic acid

## Abstract

The enhancement of plant secondary metabolite production in cell suspension cultures through biotic or abiotic elicitation has become a potential biotechnological approach for commercialization or large-scale production of bioactive compounds. *Gymnema sylvestre* R.Br. is an important medicinal plant, rich in a group of oleanane triterpenoid saponins called gymnemic acid, well known for its anti-diabetic activity. Two endophytic fungal strains were isolated from the leaves of *G. sylvestre* and identified as *Polyancora globosa* and *Xylaria* sp. based on the PCR amplification and internal transcribed spacer (ITS 1-5.8S-ITS 2) sequencing of 18S rRNA gene. The process of elicitation of cell suspension cultures of *G. sylvestre* with dried powder of fungal mycelia (DPFM) and extracellular culture filtrate (ECF) of endophytic fungi consistently enhanced the accumulation of gymnemic acid and the DPFM was proved to be an effective elicitor when compared to the ECF. The DPFM elicited the gymnemic acid content in the range of 2.57–10.45-fold, while the ECF elicited the gymnemic acid content in the range of 2.39–7.8-fold. *P. globosa*, a novel and a rare endophytic fungal strain, has shown a great influence on the production of gymnemic acid. Cell suspension cultures elicited with DPFM of *P. globosa* produced higher amount of gymnemic acid content (124.23 mg/g dried cell weight) compared to the cultures elicited with DPFM of *Xylaria* sp. (102.24 mg/g DCW). But the cultures treated with consortium of DPFM of both fungi showed great influence on the production of gymnemic acid (139.98 mg/g DCW) than the cultures treated with DPFM alone. Similarly, cultures treated with consortium of ECF of both fungi produced more gymnemic acid content (94.86 mg/g DCW) compared with cultures treated with ECF of *Xylaria* sp. (77.93 mg/g DCW) and ECF of *P. globosa* (78.65 mg/g DCW) alone.

## Introduction

Plant cell and tissue cultures are potential and alternative sources for the large-scale production of desired bioactive compounds which cannot be synthesized chemically, due to their complex structures. For this purpose, callus and cell suspension cultures are more preferred, but their yield does not match up to the growing demands. Many researchers have attempted to increase the yield of bioactive compounds through in vitro methods such as metabolic engineering, cell immobilization, optimization of nutrient medium, nutrient manipulations (Archana et al. 2012), addition of precursors and addition of elicitors (Chodisetti et al. 2015; Kiran et al. 2011).

Elicitors are defined as biofactors or chemical molecules of biotic or abiotic origin which can trigger a similar response as stress induced in the plants. Elicitors can alter the physiological or metabolic activities in the plants by triggering signal transduction pathway which in turn can increase the production of secondary metabolites. Elicitors can trigger their response by inducing the gene expression pathways. Endophytic fungi are the intriguing fungal species which live inside the healthy plant tissues without causing any apparent symptoms of disease. In this symbiotic association, many benefits from the endophytic fungi have been transferred to the host plant, which includes production of natural active compounds (Kusari and Spiteller 2011 ), production of growth promoters, tolerance to drought, secretion of defensive chemicals for protection against pathogens, pests and diseases, biochemical modifications in their host plant (Aly et al. 2011; Wani et al. 2015), enhancers or elicitors of bioactive compounds through tissue culture systems including callus and cell suspension cultures (Dass and Ramawat 2009; Kiran et al. 2011).

Triterpenoid saponins are a class of plant secondary metabolites synthesized via isoprene pathway by the cyclization of 2,3 oxidosqualene, an aglycone precursor to which one or more sugar moieties are added (Xu et al. 2004). Triterpenoid saponins are primarily divided into two groups, oleanane (β-amyrin) and dammarene type. Triterpenoid saponins possess a wide range of pharmacological activities including inhibitory activity on glucose absorption (Hideto et al. 2006), cholesterol reducing (Gurfinkel and Rao 2003), anticancer, anti-inflammatory and antimicrobial activities (Ridout et al. 1991; Netala et al. 2015). Yields of triterpenoid saponins have been successfully enhanced in experimental systems by treating the cells and tissues with different biotic and abiotic elicitors (Veerashree et al. 2012).

*Gymnema sylvestre* R. Br. is a vine plant which belongs to Asclepiadaceae family, native to India, South Asia and Africa (Ye et al. 2000). The leaves of this plant are called Gur-mar, renowned for the treatment of diabetes in India for over 2000 years (Agarwal et al. 2000). The leaves also possess lipid lowering properties (Rachh et al. 2010), hepatoprotective (Jachal 2002), anti-inflammatory (Malik et al. 2008), free radical scavenging (Ohmori et al. 2005) and antimicrobial properties (Satdive et al. 2003). All these activities are mainly due to the presence of triterpenoid saponins in the leaves of *G. sylvestre*. The total triterpenoid saponin fraction in the leaves of *G. sylvestre* is called as gymnemic acid (Ye et al. 2000). 18 different types of gymnemic acids (GA I to GA XVIII) were reported from the leaves of *G. sylvestre*. Gymnemic acid constitutes gymnemagenin as its backbone (sapogenin) with different sugar molecules attached to it (Liu et al. 1992). Gymnemic acids are differentiated based on the type and number of sugar molecules attached to gymnemagenin. The numerous types and complex structure of gymnemic acids make their chemical synthesis very difficult for large-scale production in industry.

Many researchers have reported the successful elicitation of plant bioactive compounds by the endophytic fungi through cell suspension cultures. The accumulation of diosgenin, a steroid, was elicited through cell suspension cultures by the endophytic fungus *Fusarium oxysporum Dzf17* isolated from *Dioscorea zingiberensis* (Peiqin et al. 2011). The accumulation of terpenoids, isoeuphpekinensin and euphol was elicited through cell suspension cultures of *Euphorbia pekinensis* by the endophytic fungus *Fusarium* sp E5 (Gao et al. 2011). The production of inophyllum was elicited by endophytic fungi *Nigrospora sphaerica* and *Phoma spp* through cell suspension cultures of *Calophyllum inophyllum* (Kiran et al. 2011). Endophytic fungal culture filtrate was used for the elicitation of phenyheptarin production in *Bidens pilosa* cell cultures (Di Cosmo and Misawa 1995). The production of indole glucosinolates and camelexin was elicited using endophytic fungus *Erwinia carotovora* in *Arabidopsis thaliana* cell cultures (Brader et al. 2001). *Trichoderma viridae* and *Pythium aphanidermatum* were used for the elicitation of the production of ajmalicine (Namdeo et al. 2002) and N-acetyl-tryptamine in *Catharanthus roseus* cell cultures (Eilert et al.^1986^). The accumulation of coumarin and its derivatives was elicited through cell cultures of *Petroselinum crispum* by treatment with endophytic fungal elicitor (Kombrink and Hahlbrock 1986). Abiotic elicitors such as jasmonic acid and methyl jasmonate were employed for the elicitation of phenylpropanoides and centellosides in cell suspension cultures of *Hypericum perforatum* and *Centella asiatica*, respectively (Gadzovska et al. 2007; Bonfill et al. 2011).

There were reports on the elicitation of gymnemic acid through cell suspension cultures using different elicitors including methyl jasmonate, salicylic acid (Veerashree et al. 2012), yeast extract, chitin, pectin, culture filtrates of *Escherichia coli, Bacillus subtilis, Saccharomyces cerevisiae, Agrobacterium rhizogenes* and *Aspergillus niger* (Chodisetti et al. 2013). In this study, we report the elicitation of gymnemic acid through cell suspension cultures of leaves of *G. sylvestre* using two different endophytic fungi isolated from the same plant.

## Materials and methods

### Collection of plant material

Leaves of the *G. sylvestre* were collected from medicinal plant garden, authenticated with taxonomist and the voucher specimen (SVUBHGS-050288) was deposited in the herbarium, Department of Botany, Sri Venkateswara University, Tirupati, Andhra Pradesh, India.

### Callus induction and initiation of cell suspension cultures

Callus was successfully induced from the leaves of *G. sylvestre* on MS media (Murashige and Skoog 1962) supplemented with 2.0 mg/L 2, 4-dichlorophenoxyacetic acid (2, 4-D) and 1.0 mg/L Kinetin (Netala et al. 2014). Cell suspension cultures were initiated by inoculating 2 g of callus masses in 100 ml of MS liquid media supplemented with the 2.0 mg/L of 2,4-D and 1.0 mg/L Kinetin. The cell suspensions were aerated in shaking incubator (Labline, India) at 120 rpm, at a temperature of 24 ± 2 °C with 16/8-h light/dark cycle.

### Isolation of endophytic fungi

Mature leaves of *G. sylvestre* were first washed thoroughly under running tap water and then with sterile double distilled water. Then, the leaves were treated with 70% ethanol and then washed thoroughly with sterile double distilled water. Under laminar air flow (LAF) chamber, leaves were treated with 3% NaOCl for 4 min followed by rinsing with sterile double distilled water for 3 min. Finally, leaves were surface sterilized with 96% ethanol for 30 s followed by thorough washing with sterile double distilled water. Such processed leaves were cut into segments of 2 × 2 cm size and injured before inoculation. Leaf segments were aseptically transferred to Petri plates containing potato dextrose agar (PDA) medium. Plates were incubated at 24 ± 2 °C for 8–10 days. After 10 days, fungal mycelia that grown on the surface of the PDA medium were picked and transferred to fresh PDA plates. Identification of the pure fungal cultures was carried out by molecular characterization through gene amplification and sequencing of 18S rRNA.

### Molecular characterization of endophytic fungi

#### Isolation of fungal genomic DNA

The fungal mycelium that grown on PDA was harvested placed into 1 mL Millipore water, frozen in liquid nitrogen and mechanically disrupted using mortar and pestle. Genomic DNA was isolated by HiPurA fungal DNA isolation kit (Himedia).

#### PCR amplification and sequencing of 18S rRNA gene

Fungal domain-specific primers ITS1–5′-TCCGTAGGTGAACCTGCGG-3′ (forward primer) and ITS4–5′-TCCTCCGCTTATTGATATGC-3′ (reverse primer) were used for the amplification of 18S rRNA gene from the extracted genomic DNA. Amplification was carried out in a 50 µL reaction mixture consisting of 50 ng of template DNA, 20 p mol of each primer, 0.4 U of Taq DNA polymerase, 200 μM of each dNTP and 1.5 mM MgCl_2_ in a CG palm cycler (Genetix biotech Asia). PCR cycles consisted of initial denaturation at 94 °C for 5 min followed by 30 cycles of 94 °C for 45 s, annealing at 55 °C for 1 min, extension at72 °C for 1 min and a final extension at 72 °C for 7 min. PCR amplification was confirmed by running the amplified product in 1.2% w/v agarose gel electrophoresis with ethidium bromide staining and documented using gel documentation system (Major science, UVDI). The PCR products were purified using gel extraction kit. Nucleotide sequencing was carried out using Sanger’s method at MWGAG Biotech, Bangalore, India. The homology search of the sequences obtained was carried out using BLASTn at NCBI GenBank (http://www.ncbi.nlm.nih.gov). The multiple sequence alignment of the obtained sequences and closely related sequences retrieved from NCBI GenBank was performed using ClustalW2 with default parameters. A phylogenetic tree was constructed by the Neighbor-Joining (NJ) method with nucleotide pairwise genetic distance corrections. A Bootstrap test of phylogeny was carried out to check the reliability of tree topology as a percentage of 1000 replications. All branches with <70% bootstrap support were collapsed.

#### Preparation of biotic elicitors and elicitation medium

Biotic elicitors and elicitation medium were prepared according to the method described by Kiran et al. (2011) with slight modifications. The isolated two endophytic fungal species were cultured in potato dextrose broth (PDB) and incubated at 24 ± 2 °C. Mycelia mats were developed in the culture vessels on the surface of the PDB after 12 days of incubation. The culture vessels were autoclaved at 121 °C for 10 min before harvesting the mycelial mats. Mycelia mats were harvested, filtered and collected the biomass and, extracellular culture filtrate (ECF). The collected biomass was thoroughly washed with sterile double distilled water to remove medium components. Then, the biomass was air dried and grounded to a fine powder. This fine powder is named as dried powder of fungal mycelia (DPFM). This DPFM was added to the liquid MS media with the varied concentrations ranging from 0–100 mg/100 mL medium (0, 25, 50, 75 and 100 mg/100 mL medium). The biomass-free fungal culture filtrate (ECF) was sterilized using 0.45 μM syringe filter (Millipore, USA) and then added to the media as biotic elicitor with the concentrations ranging from 0–20 mL/100 mL medium (0, 5, 10, 15 and 20 mL/100 mL medium). The DPFM of two endophytic fungal species was supplied solely and in consortium for the elicitation of gymnemic acid in the *G. sylvestre* cell suspension cultures. Similarly, the ECF of two species was added solely and in consortium for the elicitation of gymnemic acid. Biotic elicitors were added to the actively growing cell suspension cultures on day 11th of culture, i.e., a day before the content of gymnemic acid reached its maximum (Chodisetti et al. 2013). The samples for the quantification of gymnemic acid were collected at 24-h interval for 3 days, viz., 24, 48 and 72 h.

#### Gymnemic acid extraction by acid–base hydrolysis and HPLC analysis

*G. sylvestre* constitutes oleanane type triterpenoid saponins as major bioactive compounds. The total triterpenoid saponin fraction is called Gymnemic acid (Gymnemic acids I-XVIII). The different types of gymnemic acids are due to the attachment of different sugar molecules to the aglycone triterpene unit called Gymnemagenin. Due to the non-availability of difference reference standards of gymnemic acid, the quantification is usually done through the estimation of its hydrolysis product gymnemagenin.

The conversion of gymnemagenin content to gymnemic acid content was done in the following equation:

$$C = \, X \, \left( {809.0/506.7} \right)$$

where *C* is the gymnemic acid content, *X* is the gymnemagenin content, 809.0 is the molecular weight of gymnemic acid (C_43_H_68_O_14_) and 506.7 is the molecular weight of gymnemagenin (C_30_H_50_O_6_).

500 mg of dried mass of cells was taken into a 50 mL of extraction solvent (1:1 volume of ethanol and water) and dissolved completely and then 10 mL of 12% KOH solution was added and refluxed for 1 h. 11 mL of 4 N HCl was added on cooling and refluxed again for 1 h. The extract was filtered through 0.22 μM filters (Millipore, USA) and the final volume was made up to 100 mL with extraction solvent and was used for HPLC analysis. The HPLC analysis (Analytic technologies limited, India) was carried out with C18 (5.0 μM) Column using acetonitrile:water (80:20) as mobile phase at a flow rate of 1.0 mL/min and the column temperature was maintained between 26 and 27 °C. Gymnemagenin was detected at 220 nm.

### Statistical analysis

All the experiments were performed in triplicate (*n* = 3) and the data were analyzed by GraphPad Prism software and expressed as mean ± SE.

## Results and discussion

A variety of biologically active/pharmaceutically important compounds are produced by plants in the form of secondary metabolites. The application of biotic (endophytic fungus, bacteria), abiotic elicitors (metal ions, physical stress) and signaling molecules (methyl jasmonate, salicylic acid) to the plant cell suspension cultures is an effective strategy to enhance the production of secondary metabolites in vitro. *G. sylvestre*, an important medicinal plant, constitutes major bioactive compound gymnemic acid, oleanane type triterpenoid saponin. There were few reports on the elicitation of gymnemic acid through cell suspension cultures. In this study, we report the enhancement of gymnemic acid using the endophytic fungi as elicitors isolated from the same plant species.

**Fig. 1:**
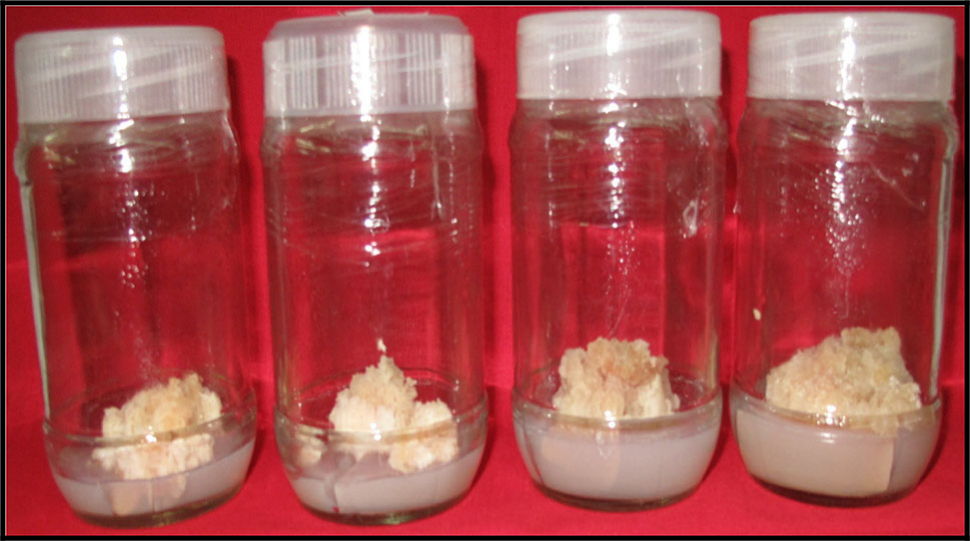
Callus cultures developed from the leaves of *G. sylvestre* on MS media containing 2.0 mg L^−1^ 2,4-D and 1.0 mg L^−1^ Kinetin

Eight different combinations of phytohormones were studied for the induction of callus from the leaf explants of *G. sylvestre*. The callus was successfully induced on MS media supplemented with 2.0 mg/L 2, 4-dichlorophenoxyacetic acid (2, 4-D) and 1.0 mg/L Kinetin (Fig. 1). Cell suspension cultures were successfully initiated by inoculating 2 g of callus masses in 100 mL.

**Fig. 2:**
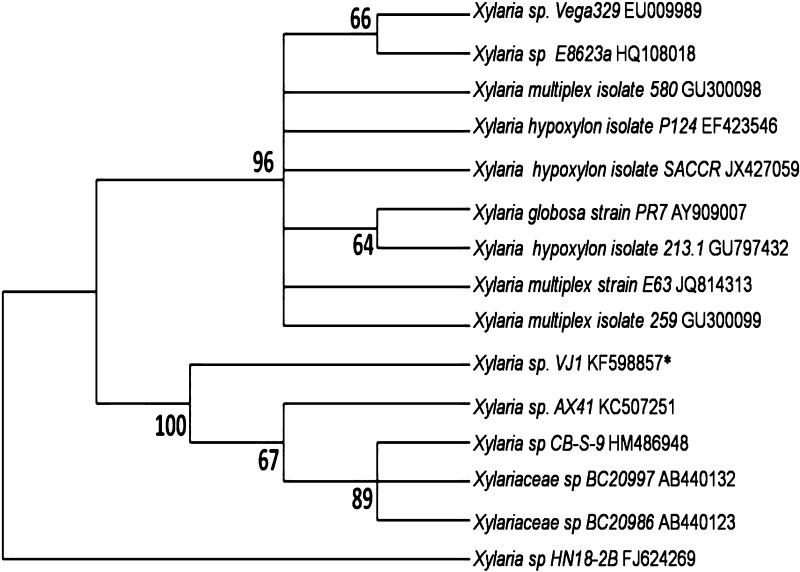
Phylogenetic tree constructed using Neighbor-joining method by comparing the ITS region of 18S rRNA gene of *Xylaria* sp. VJ1 (Accession number KF598857) with closely related strains obtained from NCBI, Genbank. The above and underneath each knot indicating the boot strap values for 1000 replications

**Fig. 3:**
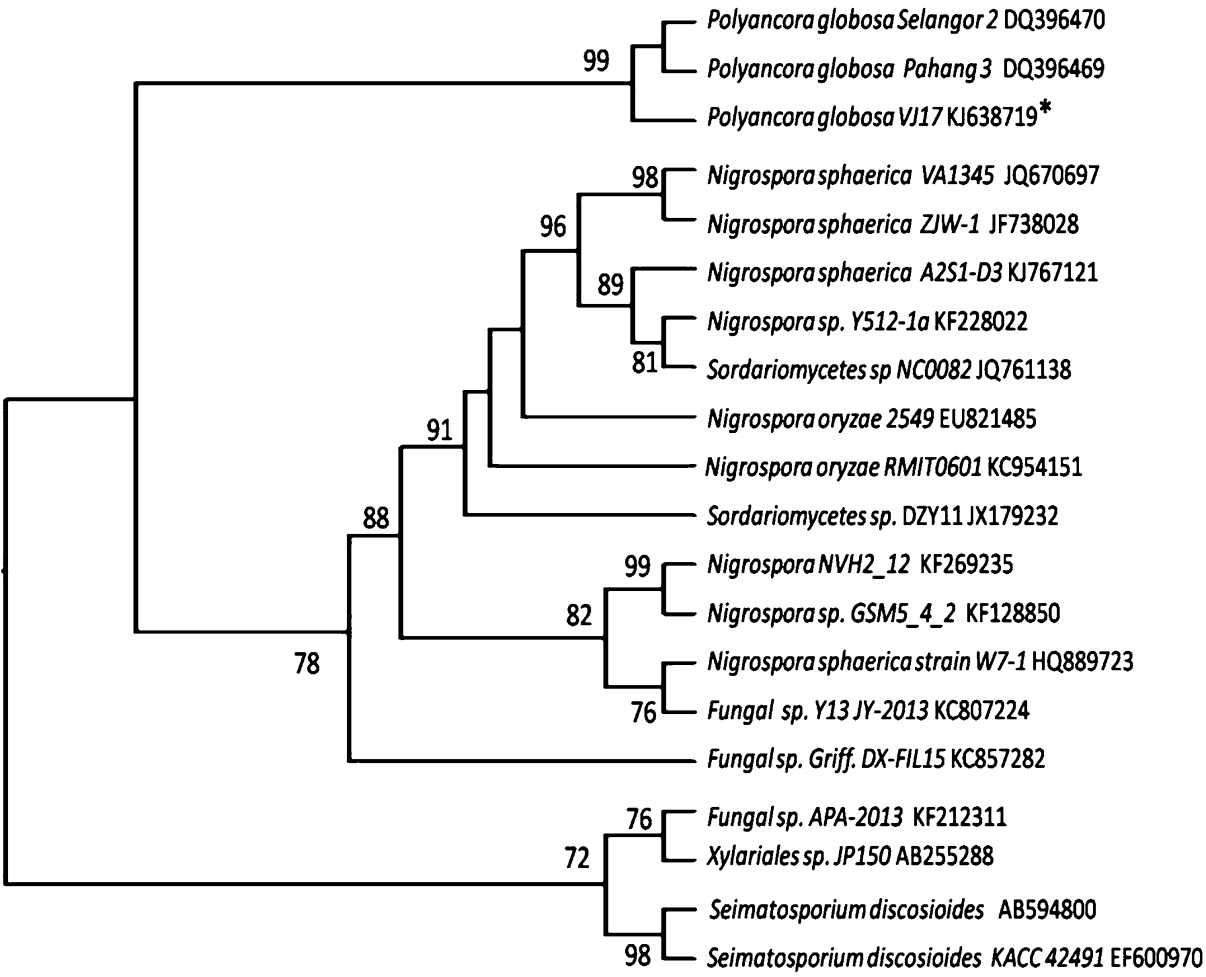
Phylogenetic tree constructed using Neighbor-joining method by comparing the ITS region of 18S rRNA gene of *P. globosa* VJ17 (Accession number KJ638719) with closely related strains obtained from NCBI, Genbank. The above and underneath each knot indicating the boot strap values for 1000 replications

Two endophytic fungi were isolated and cultured into pure isolates. Genomic DNA isolated from these two cultures was subjected to PCR for amplification of 18 S rRNA gene using ITS primers. The amplified products resulted in 518 and 541 bp sequences upon Sanger dideoxy nucleotide sequencing. The sequences obtained were subjected to BLASTn search for their homology and identification. The two fungal sequences were identified as *Xylaria* sp. and *Polyancora globosa*. The sequences were deposited in NCBI nucleotide databases and the accession numbers were assigned as KF598857 (*Xylaria* sp.) and KJ638719 (*P. globosa*). The phylogenetic trees were constructed using NJ method with the closely related sequences retrieved from NCBI Genbank. The phylogenetic tree of *Xylaria* sp. (Fig. 2) showed two clades. One clade showed nine fungal strains with 96% bootstrap support value, and the second clade showed five fungal strains with 67% boot strap supporting value. The phylogenetic tree of *P. globosa* (Fig. 3) showed three clades. One clade showed three fungal strains with 99% bootstrap support value, the second clade showed thirteen fungal strains with 78% boot strap support value and the third clade showed four closely related fungal strains with 72% boot strap support value. The correct identification of endophytic fungi by phenotypic characters is a major problem, as many of them are pleomorphic and exhibit different anamorphs *in planta* and in vitro. Hence most of the recent studies use molecular identification through ITS sequencing of 18S rRNA gene. Further, the authentification of species identified should be carried out by comparing obtained sequences with closely related sequences. In this study, the identified fungal cultures (*Xylaria* sp. and *P. globosa*) were used as biotic elicitors in the form of DPFM and ECF.

**Fig. 4:**
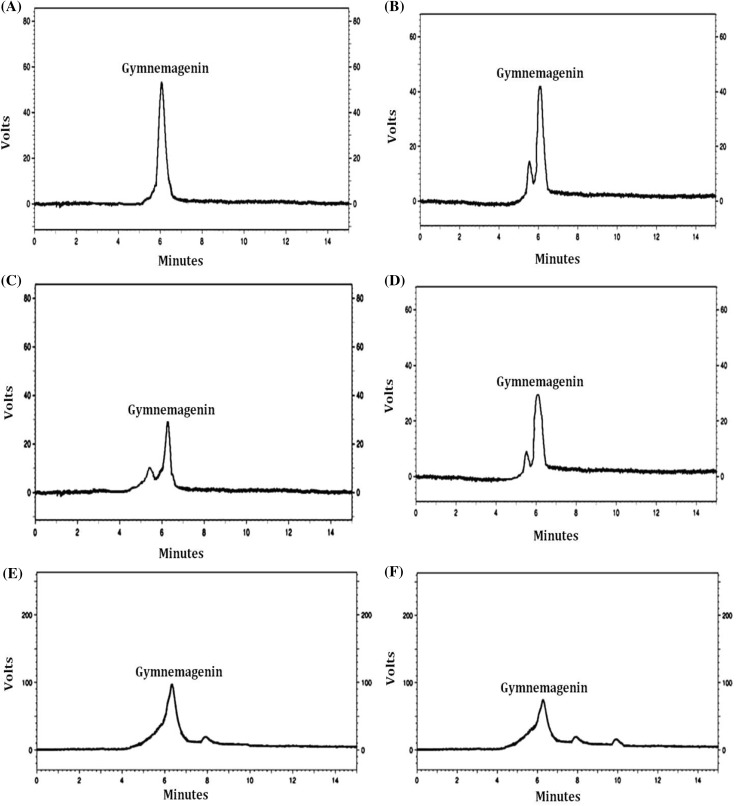
HPLC chromatograms of **a** standard gymnemagenin, **b** gymnemagenin extracted from the elicited cultures with DPFM of *P. globosa*, **c** gymnemagenin extracted from the elicited cultures with DPFM of *Xylaria* sp., **d** Gymnemagenin extracted from the elicited cultures with consortium of DPFM, **e** Gymnemagenin extracted from the elicited cultures with consortium of ECF, **f** Gymnemagenin extracted from the elicited cultures with ECF of *P. globosa*

A total of six elicitation treatments were given for the enhancement of gymnemic acid production in the cell suspension cultures of *G. sylvestre*, viz., (i) Elicitation with DPFM of *Xylaria* sp., (ii) Elicitation with ECF of *Xylaria* sp., (iii) Elicitation with DPFM of *P. globosa*, (iv) Elicitation with ECF of *P. globosa*, (v) Elicitation with consortium of DPFM of both fungi and (vi) Elicitation with consortium of ECF of both fungi. The effect of elicitors on the production of gymnemic acid was studied at 24-h interval for 3 days, viz., 24, 48 and 72 h. The analysis and quantification of gymnemic acid produced in the cell suspension cultures were carried out using HPLC as gymnemagenin equivalents by comparing with standard gymnemagenin (Fig. 4a–f).

### DPFM of *Xylaria* sp.

**Fig. 5:**
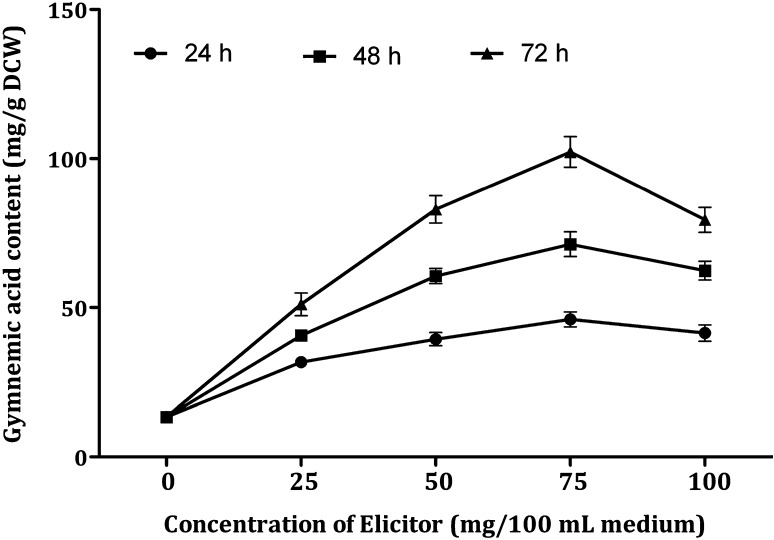
Effect of DPFM of *Xylaria* sp. with elicitation time on the accumulation of gymnemic acid in the cell suspension cultures of *G. sylvestre*

DPFM of *Xylaria* sp. was added to the cell suspensions at varying concentrations (0, 25, 50, 75 and 100 mg/100 mL medium). Figure 5 shows that all the tested concentrations enhanced the production of gymnemic acid content in the range of 2.36–7.63-fold (31.6–102.24 mg/g DCW). In this elicitation treatment, the highest amount of gymnemic acid production (102.24 mg/g DCW) was recorded after 72 h of treatment with 75 mg/100 mL of DPFM and it is 7.63-fold higher when compared to the control culture which was free of elicitor (13.4 mg/g DCW). Further increase in the concentration of elicitor from 75 to 100 mg could not enhance the accumulation, but there was a decrease (20%) in the accumulation of gymnemic acid (79.43 mg/g DCW). After 24 h of treatment, all the concentrations of elicitor increased the production of gymnemic acid in the range of 2.36–3.44-fold, but the gymnemic acid production was in the range 3.81–7.63-fold after 72 h treatment. Hence, it is indicated that 75 mg/100 mL of DPFM of *Xylaria* sp. is the optimum elicitor concentration and 72 h was determined as optimum incubation time required for the elicitor to yield maximum content.

### DPFM of *P. globosa*

**Fig. 6:**
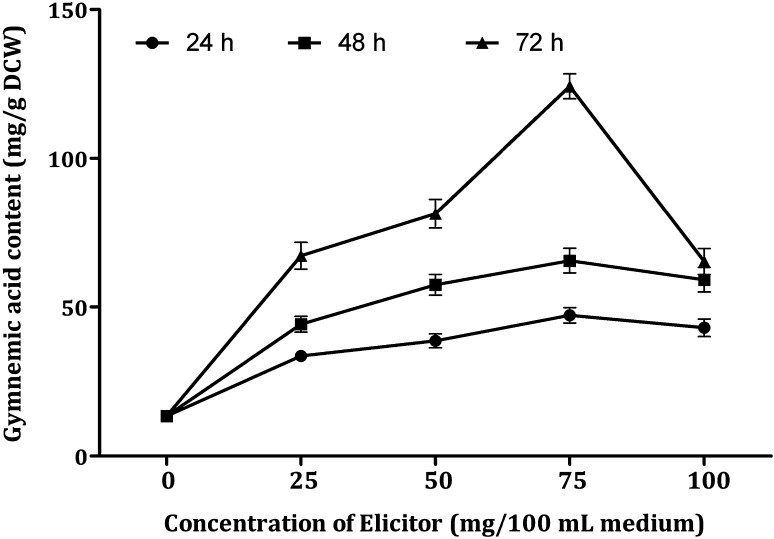
Effect of DPFM of *P*. *globosa* with elicitation time on the production of gymnemic acid in the cell suspension cultures of *G. sylvestre*

DPFM of *P. globosa* induced response towards accumulation of gymnemic acid which resulted in 2.5 to 9.27-fold enhancement in comparison to the control cultures (Fig. 6). The response towards accumulation of gymnemic acid is higher compared to elicitation with DPFM of *Xylaria* sp. (2.36–7.63-fold). Treatment with 25 mg/100 mL DPFM of *P. globosa* after 72 h incubation produced the gymnemic acid content of 67.18 mg/g DCW. Increase in the concentration of elicitor from 25 to 50 mg could not make the great impact on the production of gymnemic acid (81.34 mg/g DCW). Further increase in the concentration of elicitor from 50–75 mg induced the best response towards the gymnemic acid accumulation (124.23 mg/g DCW). When the concentration of elicitor exceeded 75 mg, there was a drastic fall (49%) in the accumulation of gymnemic acid (65.26 mg/g DCW).

### Consortium of DPFM of *Xylaria* sp. and *P. globosa*

**Fig. 7:**
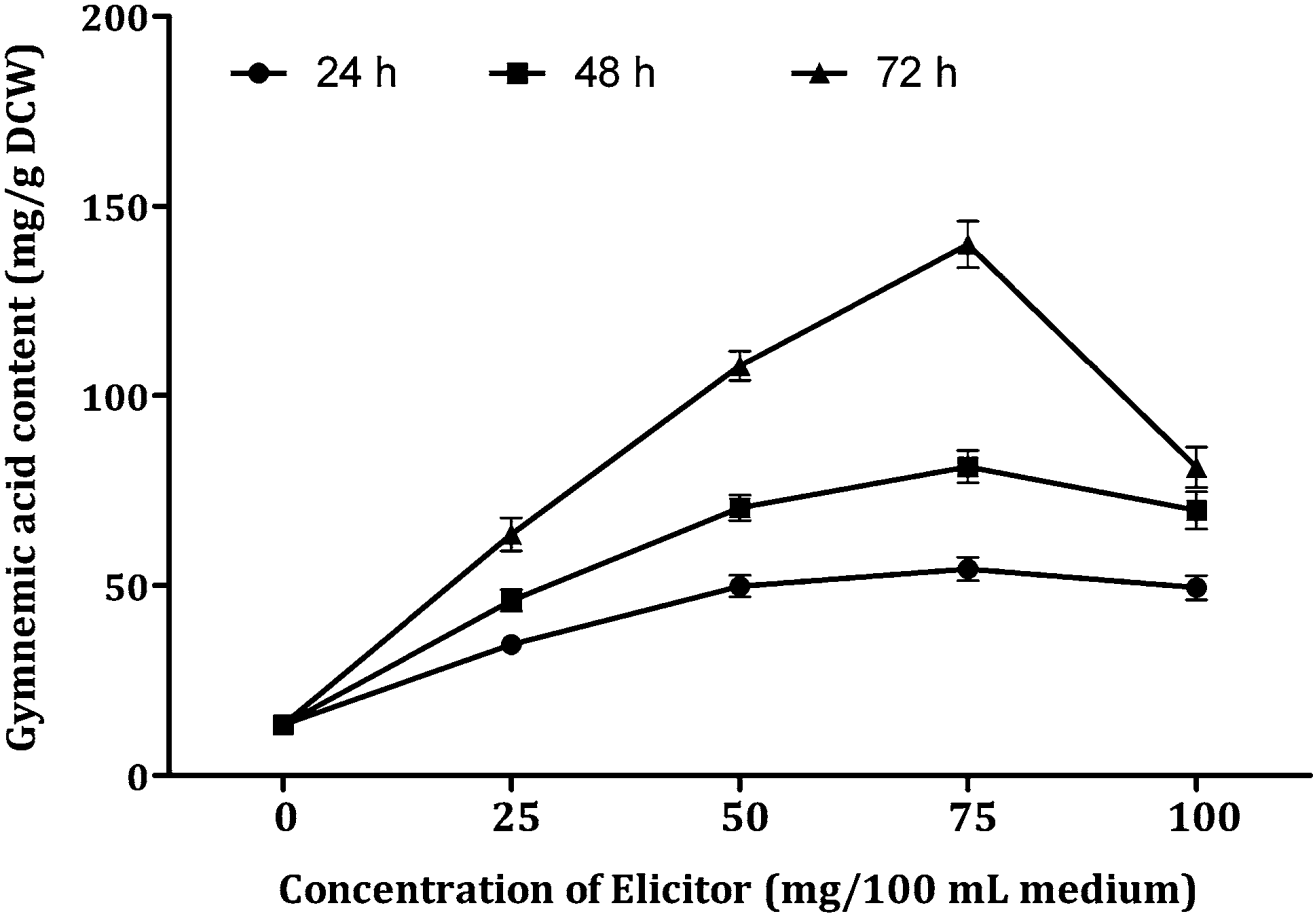
Effect of consortium of DPFM with elicitation time on the production of gymnemic acid in the cell suspension cultures of *G. sylvestre*

In the first two elicitation treatments, we studied the effect of single endophytic fungus in the form of DPFM. In this treatment, we studied the effect of consortium of DPFM of two endophytic fungi on the production of gymnemic acid in the cell suspension cultures of leaf-derived callus of *G. sylvestre*. Elicitation with consortium of DPFM enhanced the gymnemic acid content in the range of 2.57–10.45-fold compared with control cultures. Figure 7 shows the influence of consortium of DPFM on the gymnemic acid content. In the suspension cultures treated with consortium of DPFM, the highest amount of gymnemic acid was recorded after 72 h of treatment with 75 mg/100 mL (139.98 mg/g DCW). In this treatment, the yield is 10.45-fold higher compared to the control cultures that were free of elicitor. An increase in the concentration of elicitor to 100 mg could not enhance, but drastically reduced the accumulation of gymnemic acid by 43% (139.98–81.13 mg). The consortium DPFM yielded the gymnemic acid content about 1.1–1.4 times higher compared to the treatment with elicitor alone. This might be due to the synergistic effect of secondary metabolites present in the endophytic fungi.

### ECF of *Xylaria* sp.

**Fig. 8:**
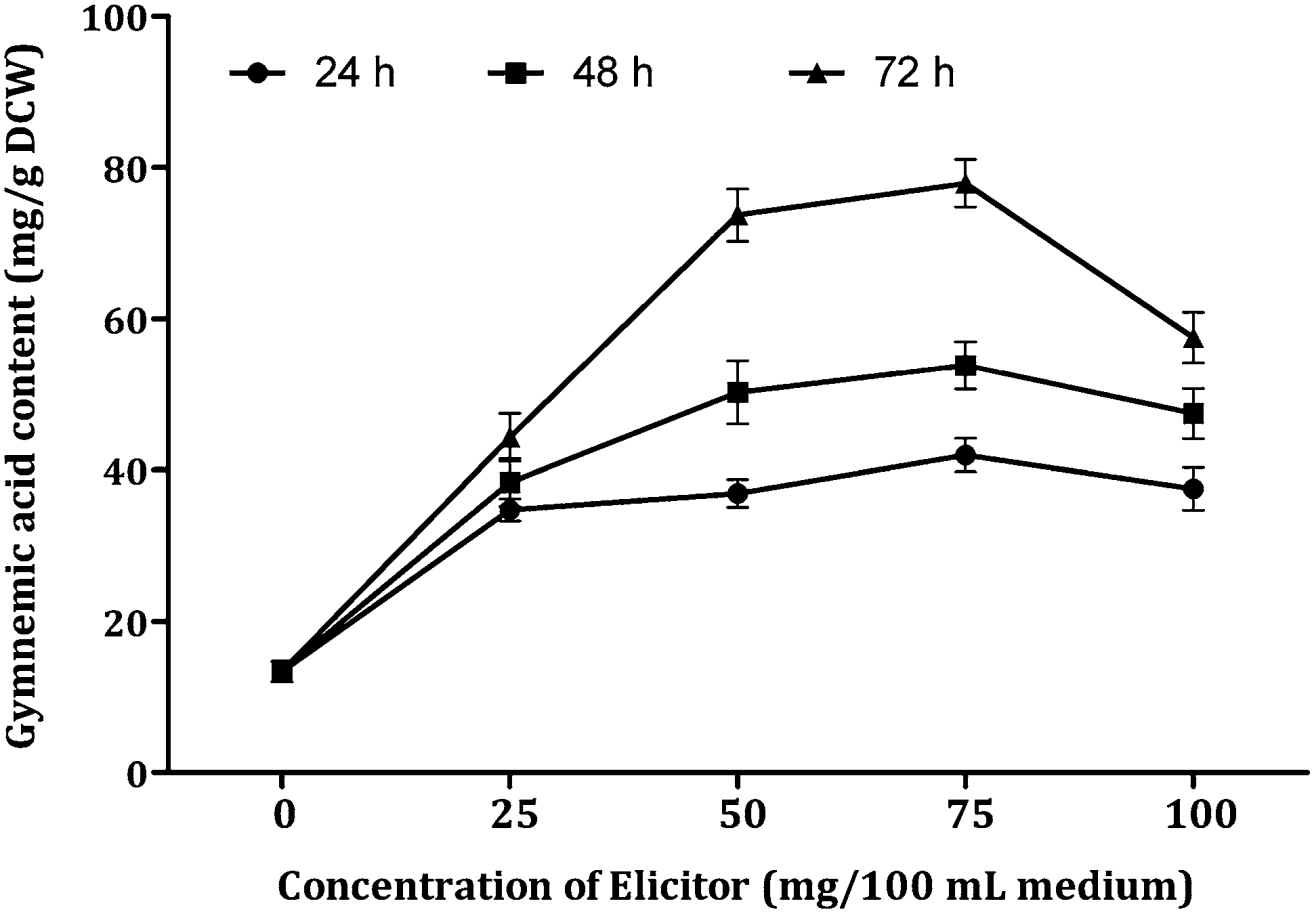
Effect of ECF of *Xylaria* sp. with elicitation time on the production of gymnemic acid in the cell suspension cultures of *G. sylvestre*

Figure 8 shows the results of the effect of ECF of *Xylaria* sp. added to the cell suspensions at different concentrations (0, 5, 10, 15 and 20 mL/100 mL medium) on gymnemic acid production. All the tested concentrations of ECF of *Xylaria* sp. increased the accumulation of gymnemic acid. In comparison to the control, 5.81-fold enhancement in the accumulation of gymnemic acid was recorded. But the amount of the gymnemic acid production with ECF of *Xylaria* sp. is lower than elicitation with DPFM of same fungus as elicitor. The highest amount of gymnemic acid was recorded after 72 h treatment (77.93 mg/g DCW) by elicitation with 15 mL of ECF. But it is lower by 1.4-fold compared by elicitation with DPFM of same fungus as elicitor (102.24 mg/g DCW). When the concentration of the ECF exceeded 15 mL, there was a drastic fall in the gymnemic acid accumulation (26.2%). The ECF of *Xylaria* sp. elicited the accumulation of gymnemic acid in the ranges 2.59–5.81-fold, whereas the DPFM of same fungi enhanced the gymnemic acid accumulation in the range of 3.81–7.63-fold and determined that DPFM was found to be suitable elicitor compared with ECF.

### ECF of *P. globosa*

**Fig. 9:**
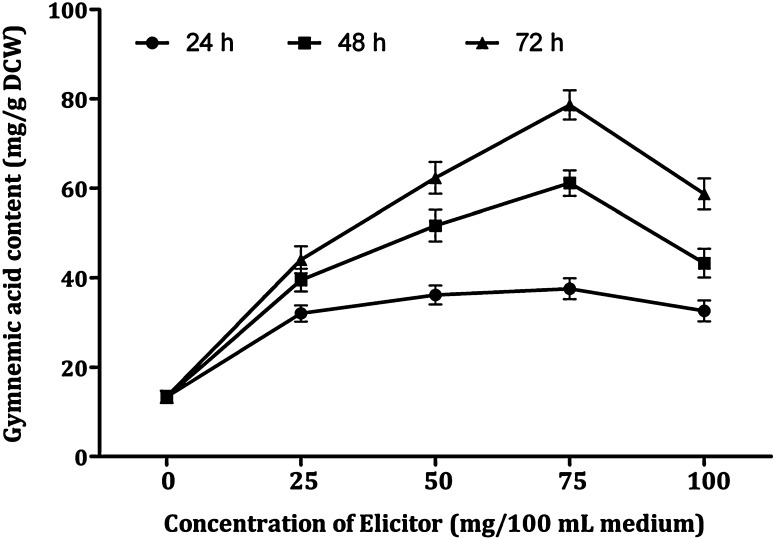
Effect of ECF of *P*. *globosa* with elicitation time on the production of gymnemic acid in the cell suspension cultures of *G. sylvestre*

All the tested concentrations of ECF of *P. globosa* elicited the production of gymnemic acid (Fig. 9). ECF of *P. globosa* enhanced the gymnemic acid production in the ranges 2.39–5.86-fold in their tested concentrations. Suspension cultures elicited with 15 mL ECF of *P. globosa* yield the highest amount of gymnemic acid (78.65 mg/g DCW) after 72 h treatment. It was followed by elicitation with 10 mL ECF (62.35 mg/g DCW). The increase in the concentration of elicitor from 15 to 20 mL of ECF could not enhance the production of gymnemic acid, but reduced the accumulation of gymnemic acid by 25% (78.65–58.75 mg/g DCW).

### Consortium of ECF of *Xylaria* sp. and *P. globosa*

**Fig. 10:**
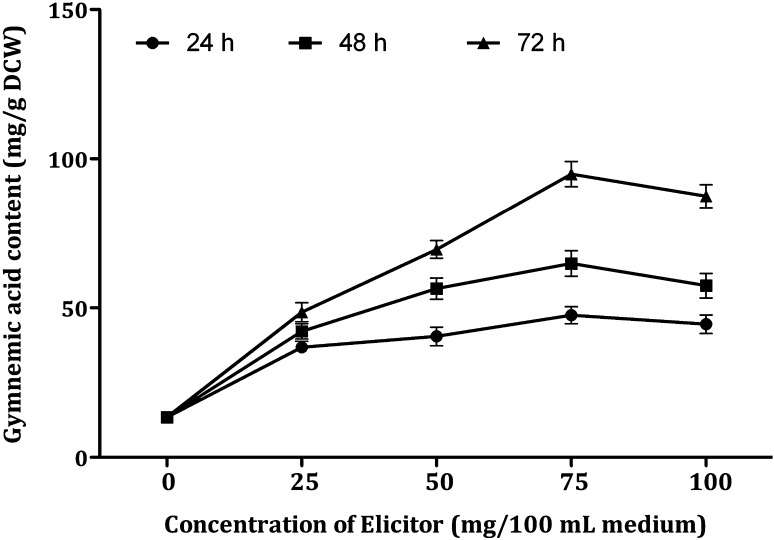
Effect of consortium of ECF with elicitation time on the production of gymnemic acid in the cell suspension cultures of *G. sylvestre*

Figure 10 shows that consortium of ECF enhanced the gymnemic acid production in the ranges 2.75–7.08-fold compared to control cultures. Treatment with 5 mL/100 mL ECF after 72 h incubation produced the gymnemic acid content of 48.54 mg/g DCW which is 3.63-fold higher compared to control cultures. Elicitation with 10 mL/100 mL ECF after 72 h incubation produced the gymnemic acid content of 69.58 mg/g DCW, which is 5.19-fold higher, compared to control cultures. The highest amount of gymnemic acid (94.86 mg/g DCW) was recorded after 72 h treatment period with 15 mL ECF as elicitor. The yield is 7.08-fold higher compared to control cultures. Increase in the concentration of elicitor to 20 mL could not enhance, but there was slight fall in the accumulation of gymnemic acid by 7.8% (94.86–87.43 mg/g DCW).

**Fig. 11:**
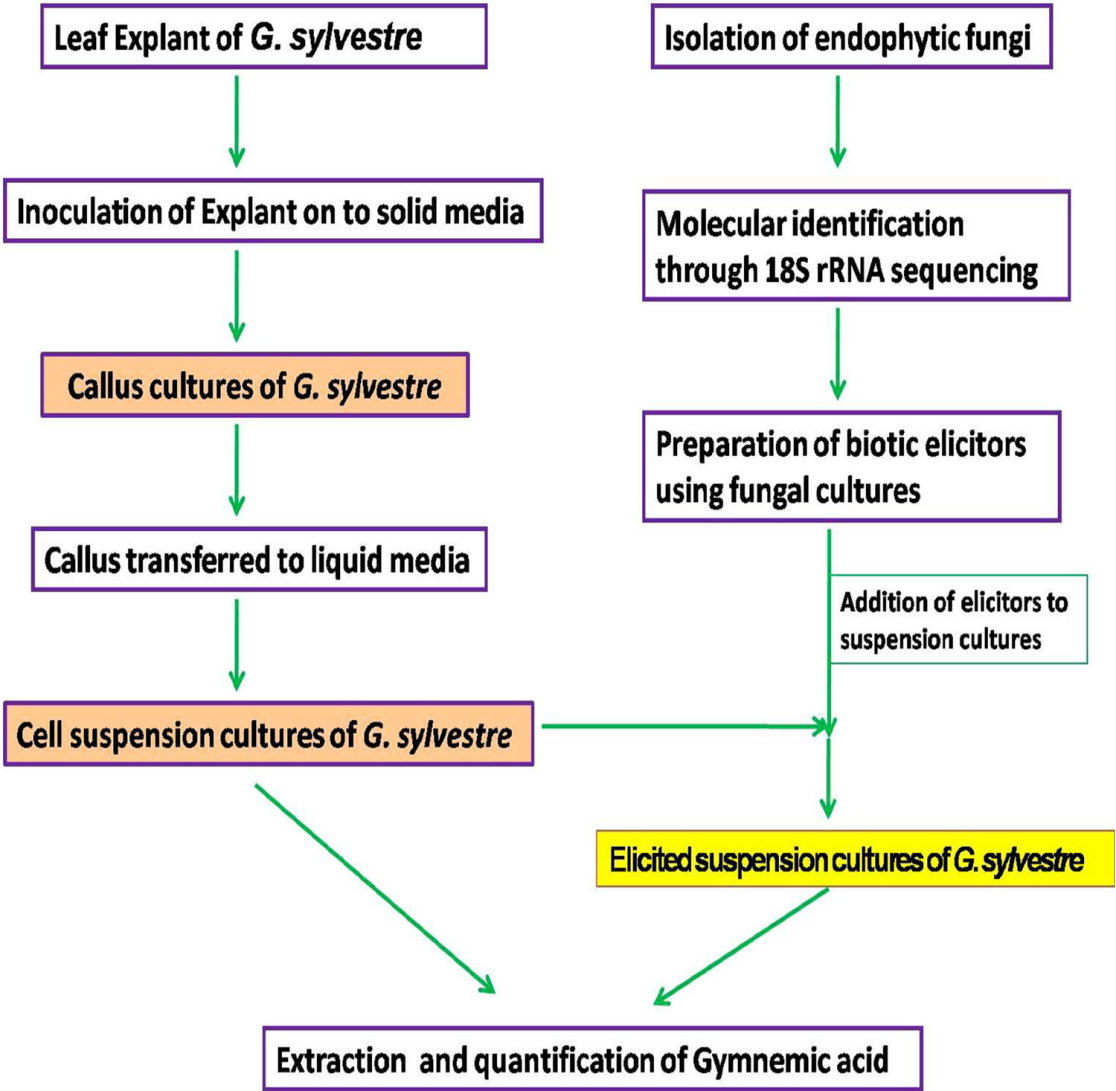
Schematic representation of the elicitation of gymnemic acid through cell suspension cultures of *G. sylvestre* using endophytic fungi

The schematic representation of the elicitation of gymnemic acid by endophytic fungi via cell suspension cultures is represented in Fig. 11.

The application of biotic or abiotic elicitors is an effective biotechnological approach to enhance the production of secondary metabolites in vitro. In the present study, we reported the influence of endophytic fungi (biotic elicitor) on the production of gymnemic acid in cell suspension cultures of *G. sylvestre*. The biotic elicitors were added to the cell suspension cultures in the form of DPFM and ECF. Elicitation of cell suspension cultures of *G. sylvestre* with DPFM and ECF of both endophytic fungi successfully enhanced the production of gymnemic acid. DPFM of endophytic fungi enhanced the gymnemic acid production in the ranges 2.36–10.45-fold, while cell suspensions treated with ECF of endophytic fungi enhanced the production of gymnemic acid in the ranges 2.39–7.8-fold. The highest amount of gymnemic acid recorded with DPFM was 139.98 mg/g DCW, while the highest amount recorded with ECF was 94.86 which is 33% less compared to the yield of cell suspensions treated with DPFM. Hence, the DPFM of endophytic fungi was proved as a very effective elicitor compared to ECF of endophytic fungi.

Among the six elicitation treatments studied, the gymnemic acid production was in the order of Consortium of DPFM of both fungi > DPFM of *P. globosa* > DPFM of *Xylaria* sp. > Consortium of ECF of both fungi > ECF of *P. globosa* > ECF of *Xylaria* sp. In all the elicitation treatments with DPFM of endophytic fungi, 75 mg/100 ml was found to be an optimum elicitor concentration required to evoke the maximum response. Similarly, in all the elicitation treatments with ECF, 15 ml/100 ml was proved to be optimum elicitor concentration to evoke maximum response. In all the elicitation treatments, the maximum amount of gymnemic acid was recorded only after 72 h of elicitation treatment. Hence, 72 h was determined as the optimum incubation time required for the elicitor to evoke maximum response.

Many efforts were made for the elicitation of gymnemic acid through cell suspension cultures, using the method of through biotic or abiotic elicitation. Veerashree et al. (2012) have reported the elicitation of gymnemic acid through cell suspension cultures by the biotic elicitors such as yeast extract, chitin, pectin and methyl jasmonate. Devi et al. (2012) have reported the successful elicitation of gymnemic acid in cell suspension cultures by biotic elicitor, *Xanthomonas* sp. Chodisetti et al. (2015) have reported the enhancement of gymnemic acid in the suspension cultures of *Gymnema sylvestre* using the signaling molecules—methyl jasmonate and salicylic acid. Chodisetti et al. (2013) have improved gymnemic acid production in the suspension cultures through biotic elicitation by *Agrobacterium rhizogenes, Saccharomyces cerevisiae, Aspergillus niger, Bacillus subtilis* and *Escherichia coli.*

In this report, we have demonstrated a strong influence of *P. globosa*, a very rare endophytic fungus on the accumulation of gymnemic acid and *Xylaria* sp. was also found to be a good elicitor. The enhancement in gymnemic acid production in this study was found to be higher than gymnemic acid levels in the earlier efforts of the biotic elicitation in cell suspension cultures of *G. sylvestre.* Compared with the fungal elicitors from other sources, endophytic fungi from the host plant (endosymbionts) can easily adapt to the in vitro cultural conditions due to their mutualistic interactions with host, which leads to enhanced growth or high differentiation of host cells which in turn leads to the production of more secondary metabolites. The enhancement in secondary metabolite production could be also due to the presence of glycoproteins, lipopolysaccharides, growth-promoting hormones and many other fungal derived compounds.

## Conclusion

In this study, we have isolated two different endophytic fungal species *Xylaria* sp. and *P. globosa* from the leaves of *G. sylvestre*. The isolated endophytic fungi were exogenously supplied for the elicitation of gymnemic acid production in the cell suspension cultures of leaf-derived callus of *G. sylvestre. P. globosa* is a very rare and novel endophytic fungus which showed very strong influence on the accumulation of gymnemic acid in the cell suspension cultures of *G. sylvestre*. A very-well known *Xylaria* sp. was also proved to be a good elicitor. In this study, the form of elicitor (biomass/extracellular filtrate) that was best suited for gymnemic acid production was also reported. Culture conditions like optimum elicitor concentration and optimum incubation time were also determined for the endophytic fungal elicitors to evoke the maximum response. To the best of our knowledge, *P. globosa* is a very rare and novel endophytic fungus and it was proved to be an effective fungal elicitor for the enhancement of bioactive compounds. A Consortium of elicitors further improved the amount of bioactive compounds compared with an elicitor alone.
